# Gonadal Atresia, Estrogen-Responsive, and Apoptosis-Specific mRNA Expression in Marine Mussels from the East China Coast: A Preliminary Study

**DOI:** 10.1007/s00128-022-03461-2

**Published:** 2022-01-24

**Authors:** Jingmin Zhu, Jiana Li, Emma C. Chapman, Huahong Shi, Corina M. Ciocan, Kai Chen, Xiaodong Shi, JunLiang Zhou, Peiying Sun, Yueyao Zheng, Jeanette M. Rotchell

**Affiliations:** 1 State Key Laboratory of Estuarine and Coastal Research, East China Normal University, Shanghai, 200062 China; 2 Department of Biological and Marine Sciences, Hardy Building, University of Hull, Cottingham Road, Hull, HU6 7RX UK; 3 School of Pharmacy and Biomolecular Sciences, University of Brighton, Lewes Road, Brighton, BN2 4GJ UK

**Keywords:** Mytilus, Estrogen, Atresia, Apoptosis

## Abstract

This preliminary survey analysed mussel atresia incidences, estrogen-responsive and apoptotic-specific molecular end points, and aqueous and gonadal levels of selected estrogens from the East China coast. Estrogen levels were low (e.g. < LOD-28.36 ng/L, < LOD-3.88 ng/g wet weight of tissue for BPA) relative to worldwide freshwater environments, but high oocyte follicle atresia incidences (up to 26.6%) occurred at selected sites. Expression of estrogen-responsive *ER2* was significantly increased in males relative to females at sites with high atresia incidences in females. A second estrogen-responsive gene, *V9*, was significantly increased at two sites in April in females relative to males; the opposite was true for the remaining two sites. Apoptosis-specific genes (*Bcl-2*, *fas*) showed elevated expression in males relative to females at the site with the highest atresia incidence. These results provide coastal estrogen levels and the utility of several estrogen-specific molecular-level markers for marine mussels.

**Supplementary Information:**

The online version contains supplementary material available at 10.1007/s00128-022-03461-2.

Studies have highlighted that water, sediment and mussels from the Shanghai area of the East China coastal region contain significantly higher levels of legacy and emerging contaminants relative to other sites in China (Table S1). Cumulative evidence suggests that the Changjiang Estuary, and the east coastline of China, receives many contaminant classes similarly to coastlines worldwide (Atkinson et al. 2003; Koyama et al. 2013; Emnet et al. 2015), and the biological implications are unknown.

Mussels, Mytilus sp. concentrate contaminants in their tissues and are widely used in toxicology studies and as bioindicator species (Beyer et al. 2017). Molluscs contain vertebrate-like steroids, such as androgens (testosterone), estrogens (estrone E1 and estradiol E2), and progestins (Reis-Henriques et al. 1990; Zhu et al. 2003) and have enzymes typically involved in the steroidogenesis pathways (Janer and Porte, 2007). The presence of sex steroids is therefore established in molluscs, but their biological role is undecided (Scott, 2013). A 10-day short term exposure to synthetic estrogen 17-α ethinyl estradiol (EE2) and E2, resulted in a significant increase in estrogen receptor (*ER2*) mRNA expression in the gonad of *M. edulis* during early stages of gametogenesis (Ciocan et al. 2010). Gonad egg yolk protein VTG mRNA expression was also significantly increased in these estrogen-exposed mussels (Ciocan et al. 2010). In parallel studies, *serotonin receptor, cyclooxygenase* and *vitelline envelope zona pellucida domain 9* (*V9*) mRNA expressions have also been impacted by E2 exposure in mussels (Cubero-Leon et al. 2010; Ciocan et al. 2011). Cumulatively, these studies suggest that mussels are susceptible to exogenous sources of estrogens. In terms of the subsequent cellular and tissue level pathological impacts of endocrine disrupting chemicals (EDCs) in bivalve populations in general, there have been reports of intersex (Ortiz-Zarragoitia and Cajaraville 2010), gonadal neoplasms (Barber 2004), atresia (Ortiz-Zarragoitia et al. 2011) and apoptosis (Matozzo and Marin 2005). The latter results from programmed cell death and is part of molluscan immune defence; the extrinsic apoptosis pathway involves death receptors (fas, Trail, TNF) and G-protein coupled receptors, whereas the interlinked intrinsic pathway is controlled by Bcl-2 family proteins (Kiss 2010).

The aim of this study was to perform a pilot investigation into the reproductive health of marine mussels, Mytilus sp., comprising either *M. coruscus* or *M. galloprovincialis* (Qu et al. 2019; Ding et al. 2020) from the East China coastal region, at four sampling locations and for two seasons at one site (to indicate any seasonal variation). Biomarkers were adopted for biomonitoring in marine molluscs including; targeted molecular endpoints responsive to estrogenic compounds (*V9* and *ER2* mRNA expression) (Ciocan et al. 2010) and specific to apoptosis (*Bcl-2* and *fas*) (Lee et al. 1997; Morita et al. 1999). These were used alongside histology to identify atresia as a relevant biological endpoint of impact relating to reproductive health (Smolarz et al. 2017) as well as aqueous and gonadal tissue estrogen concentrations to determine exposure levels and uptake.

## Materials and Methods

Mytilus sp*.* (n = 26–60, depending on availability) were collected at low tide from four Chinese coastline locations: Qingdao (comprising 4 local subsampling sites QD-A, QD-B, QD-C and QD-D), Yantai (YT), Shengsi (SS) and Xiamen (XM), to investigate spatial variation, and during two seasons at one sampling location (Qingdao) to investigate any temporal variation. During April 2014, samples were collected at QD-A to QD-D. During July 2014, samples were collected from YT, QD-B, QD-D, SS and XM (Fig. S1, Table S2). Mussels (n = 26–60) were measured and gonad tissues were immediately dissected into 0.5 cm^2^ pieces: one was fixed in 4% formaldehyde and stored at room temperature for histology (for which n = 1 slides for each individual was analysed), one was kept in RNAlater™ (Sigma-Aldrich, USA) at − 20°C for molecular analyses, and one was stored at −80°C until chemical analyses. 3 L of seawater was taken from each site and stored at − 20°C until chemical analyses. Samples (Table S2) were also analysed blind (no knowledge of sex or development stage) for gene expressions using mussels from each site (and at QD from two seasons). Total RNAs were extracted from ~ 20 mg of each mussel gonad with RNeasy reagents (Qiagen, Germany) with a DNase I digest. rRNA integrity was determined by 1% agarose-formaldehyde gel electrophoresis. First strand cDNAs were generated from 1 μg total RNA using PrimeScript™ RT reagents (TaKaRa, Dalian, China). Real-time PCR reactions (final volume 20 μL) contained 10 μL SYBR® Premix Ex Taq, 2 μL cDNA, 0.4 μM primers, and 0.4 μL ROX Reference Dye (Takara, Dalian) (Table S3). Reference genes *18S* and *EF1a*, previously validated for stability in estrogen-exposed mussel gonads (Cubero-Leon et al. 2012), were selected for relative quantification. A control without cDNA was used to determine the specificity of target cDNA amplification. Cycling parameters were: 95°C for 30 s, 40 cycles of 5 s at 95°C and 34 s at 60°C with a 7500 RT PCR system (Applied Biosystems, U.S.A.). Melting curves and gels confirmed specificity of amplified products. The efficiency of each primer pair was calculated by cDNA dilution factors. Relative expression levels of the four target genes were calculated using the comparative ΔCT method (Livak and Schmittgen 2001), outliers were defined as twice the standard deviation. Mussel gonads were wax embedded, transversely sectioned (6 μm), stained with haematoxylin–eosin, and observed with an Olympus BX53 microscope (Japan) to determine the sex (where possible), stage of maturation (Seed 1969), and occurrence of atresia.

Aqueous stocks (1000 mg/L) of estrogen standards (E1, E2, E3 and EE2) and the internal standard (E2-d2) (Dr. Ehrenstorfer™, LGC Ltd) were diluted with methanol (10 mg/L). All solvents were HPLC grade. Water samples were extracted as described by Yan et al. (2013) and Shi et al. (2014). Briefly, water samples were filtered using pre-ashed 0.7 μm GF/F filter and spiked with 20 ng of internal standards. Water samples were pre-conditioned with ultra-pure water and methanol and passed through an Oasis HLB cartridge at a standard flow rate (5–10 mL/min) for solid phase extraction (SPE). The compounds were eluted with 10 mL of methanol then concentrated to 0.5 mL. Mussel gonads (1.5 g) were injected with 20 ng internal standards and extracted with an ASE 350 accelerated solvent extractor by a mixture of methanol and acetonitrile (1:1). The targeted EDCs were analyzed by a Waters Acquity™ UHPLC–MS/MS system according to Ye et al. (2013). Targeted EDCs were measured in negative ion mode. The flow rate of the desolvation gas (N2) was 800 L/h, and temperature was set at 500°C. The flow rate of the collision gas (Ar) was 10.2 mL/h, and capillary voltage was 2.8 V. The limit of quantification (LOQ) and limit of detection (LOD) in aqueous samples were 0.30–1.97 ng/L and 0.10–0.49 ng/L respectively, and 0.33–1.55 ng/g wet weight and 0.15–0.44 ng/g respectively in tissue samples.

Statistical analyses were performed using SPSS. The Kolgomorov-Smirnov test was used to examine normality of residuals and the homogeneity of variance. Sex ratio bias was determined using Chi squared test (*p* < 0.05). For the mRNA expression data, the Scheirer-Ray-Hare (SRH) test was used for QD sites in April to examine the difference caused by sex and sampling site and determine if the two factors interact. For all sites, significance for relative gene expression between sexes at individual sampling sites or the same sex at different sampling seasons, was tested using the Kruskal–Wallis (KW) non-parametric test (non-normal distribution). Outliers, according to the MIQE guidelines (Bustin et al. 2009), were excluded from the statistical analysis. Significance was accepted at: * = *p* < 0.05, ** = *p* < 0.01, *** = *p* < 0.001.

## Results and Discussion

**Fig. 1:**
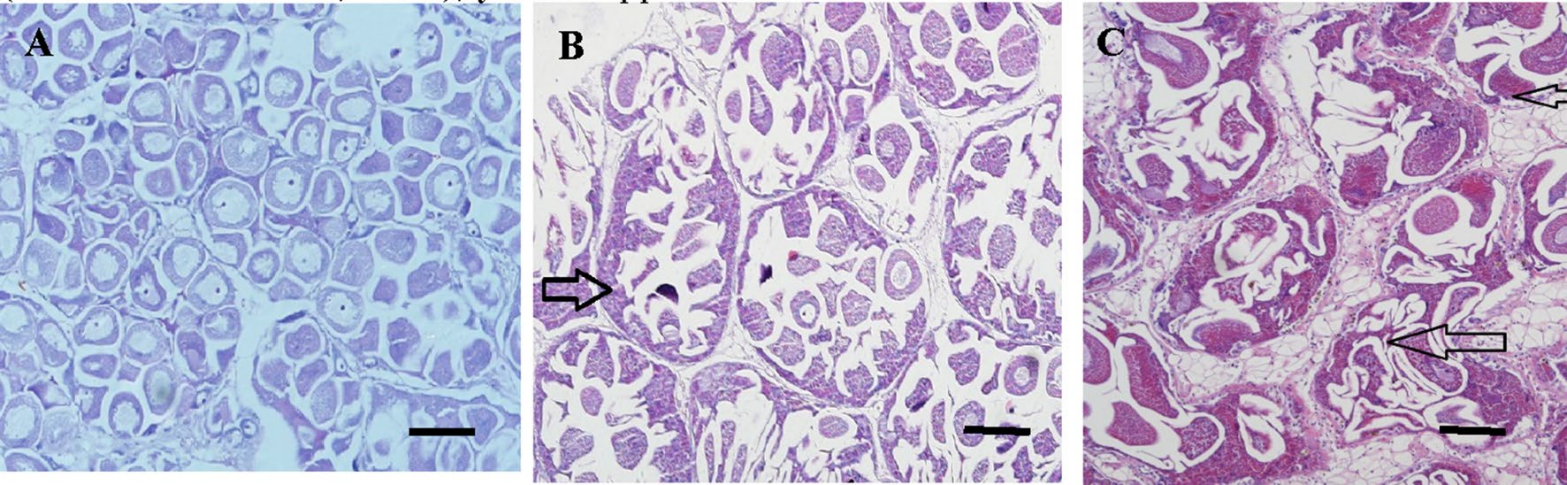
Mytilus sp. gonads stained with H & E stain. **A** normal female at developing/mature stage (βIII/βIV); **B** mature female (βV/γIV) with atretic oocytes (arrows); **C** massive degeneration (atresia) of female follicles in a mature gonad. Size bar 100 µm, 200 × magnification, (Seed, 1969)

Following histological examination, sex and stage of gametogenesis were determined, as well as the incidence of atresia in females only (Table S2, Fig. 1). A sex ratio bias in favour of males was observed at QD-D (relative to QD-A to C) in April (Table S2). Many spent mussels were detected in July, prohibiting sex ratio calculations. No previous marine mussel sex ratio data is available for this region, though female sex ratio bias has been reported in mussels impacted by spilled oil in the Bay of Biscay (Ortiz-Zarragoitia et al. 2011), and in clams, *Scrobicularia plana*, at selected sites from the English Channel (Pope et al. 2015). A male bias has previously only been observed in *S. plana* from six locations in the English Channel region (Pope et al. 2015) and following a > 36 week laboratory exposure using the bivalve *Gomphina veneriformis* to tributyltin (Park et al. 2015). Herein Mytilus sp. were observed at various stages of gametogenesis (Table S4), a process that is seasonal and spatially dependent, and appears to reflect the variability in gametogenesis stages characteristic of the species (Seed and Suchanek 1992), yet the apparent occurrence of a sex ratio bias is unusual.

Atresia is a natural part of the gametogenesis cycle in which the ovarian follicles die, allowing the resorption of gametes at the end of the hatching stage, and a resting period before a new cycle begins. Pre-spawning oocyte atresia may also occur (Beninger 2017). A typical indicator of atresia in females is vacuolisation within eggs (Fig. 1B). The incidence of oocyte atresia varied temporally and spatially; atresia was detected in females at all QD sites in April, with the highest incidence (26.6%) observed at QD-B, yet no atresia was found in females at any sites in July (Table S2). An increased incidence of atresia has previously been reported in *M. provincialis* as a natural occurrence in the winter in Galicia, Spain as a result of unfavourable conditions for spawning after the gametes ripened (Suarez et al. 2005). High incidences of atresia have also been reported in *M. edulis* from Boston Harbor/Cape Cod Bay, U.S.A. (Kimball 1996), and *M. trossulus*, Gulf of Gdansk, Poland (Smolarz et al. 2017), as well as following experimental exposure of *M. edulis* to North Sea oil and alkylphenol (Aarab et al. 2004), metals using *Crassotrea angulata* (Vaschenko et al. 2013), and estrogens using *M. trossulus* (EE2, at 50 and 500 ng/dm^3^) (Smolarz et al. 2017). In vertebrates, an increased incidence of oocyte atresia has also been reported in fish (*Clarias gariepinus* and *Chalcalburnus tarichi*) experimentally exposed to various estrogens (EE2 at 50 ng/L and 100 ng/L) (Kaptaner and Unal 2011; Sridevi et al. 2015). Exposure of medaka fish (*Oryzias latipes*) to 10 ng/L EE2 failed to induce atresia though did increase the rate of apoptosis in testicular cells (Weber et al. 2004).

**Fig. 2:**
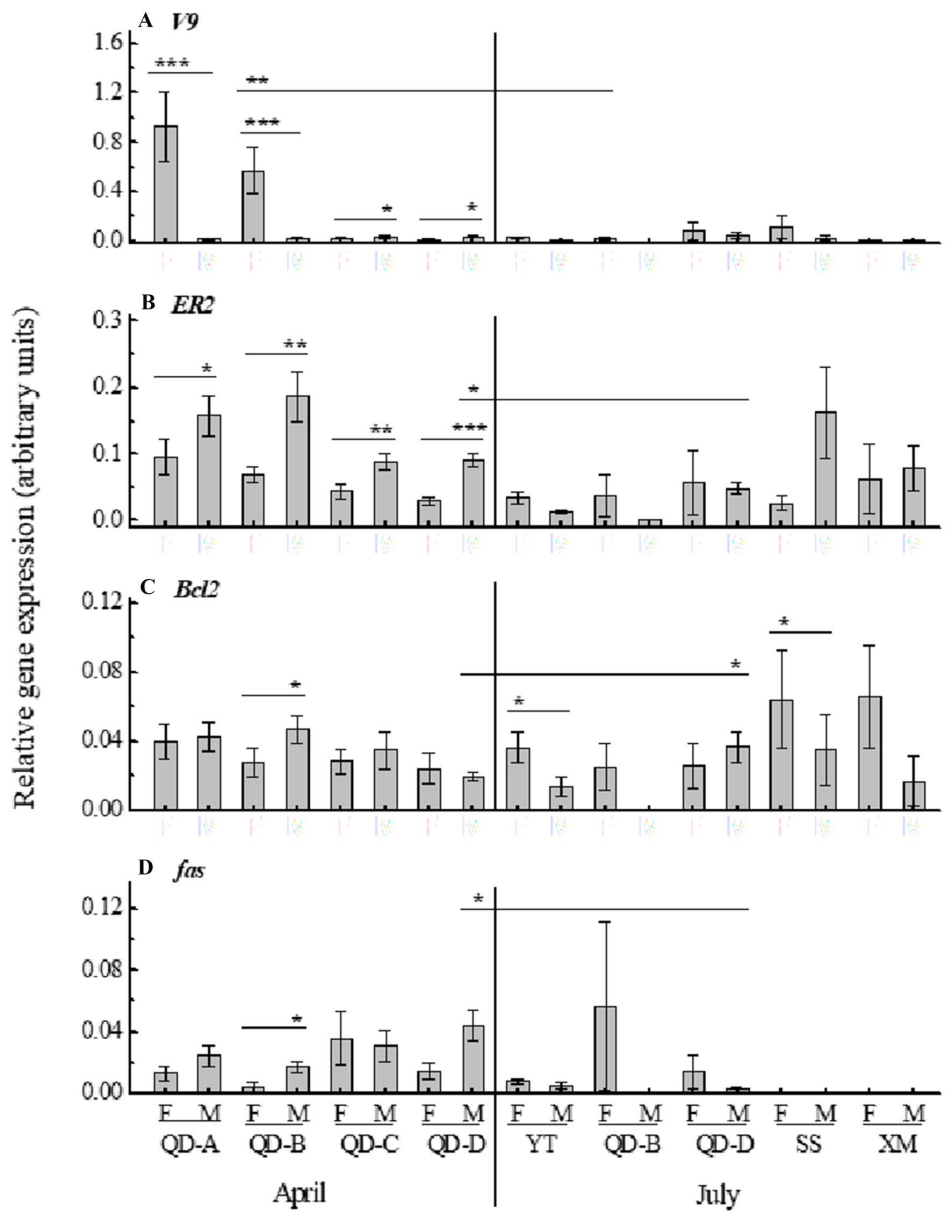
Relative gene expression of target genes) *V9*, **B** *ER2*, **C** *Bcl-2*, and **D** *fas* in mussel gonad tissues taken from seven geographical sampling sites and two sampling seasons. Data are plotted as mean ± SEM (n = variable, Table S2). Lines above the bars denote significant differences (Kruskal–Wallis test): *p < 0.05, **p < 0.01, ***p < 0.001. Abbreviations of sampling sites: *QD* Qingdao, *SS* Shengsi, *XM* Xiamen, *YT* Yantai

Gene expression was investigated as follows: broadly across four East China Sea coastal locations; more locally within one location (four subsampling sites); and finally, also at the latter location, across two seasons (spring and summer) when the natural gametogenesis cycle in molluscs is at different stages. qPCR revealed that sex influences *V9* expression levels significantly (SRH, *p* = 0.000) at QD sites in April but subsampling location (QD-A to D) does not; there was no interaction between sex and site. Female mussels at QD-A and QD-B displayed elevated *V9* expression compared with males (Fig. 2A, Table S4). *V9* expression in QD-B females was significantly higher in April than July, coinciding with the early developing and mature stages of gametogenesis (Table S4, Fig. 1A). Uncharacteristically for males, which do not normally produce eggs, *V9* expression was elevated in males compared to females at QD-C and QD-D during April (Fig. 2A), indicating induction of egg-specific cellular signalling pathways. Natural seasonal variation in egg yolk associated proteins (and associated gene expression) occurs in scallop (*Patinopectin yessoensis*) and mussel with peaks in March/April coinciding with development and mature stages of gametogenesis and lower levels in summer coinciding with post-spawning/degeneration (Osada et al. 2003; Ciocan et al. 2010). Male mussels typically display a low background level of egg-related gene expression (Ciocan et al. 2010), yet males at QD-C and QD-D during April show significantly elevated *V9* expression compared with females (Fig. 2A). Similar increases in egg yolk (specifically *vitellogenin*, *VTG*) and membrane (*choriogenin/zona radiata*) gene expressions and protein levels in males have been reported in fish (*Oryzias melastigma)* and are utilised as biomarkers following estrogen: E2, EE2 and BPA (Chen et al. 2008), and xenoestrogen: refinery oil exposure (Arukwe et al. 1997).

For *ER2* mRNA expression, both sex (SRH, *p* = 0.000) and sampling site (*p* = 0.001) influence expression levels significantly in April. Expression was significantly higher in QD-D males in April compared to July (Fig. 2B). In addition, *ER2* is significantly increased in males compared with females at all QD sites in April (Fig. 2B), with a similar, though non-significant, trend observed at SS in July. The precise underlying mechanism resulting in the increase of *ER2* expression in male mussels, relative to females, is unclear due to the ongoing debate surrounding the functional role of ERs in bivalves (Scott 2013; Nagasawa et al. 2015). *V9, ER2* and *VTG*/VTG, have previously been recognized as up-regulated genes in *M. edulis* (Ciocan et al. 2010; Nagasawa et al. 2015), scallop *P. yessoensi*s (Osada et al. 2003), and oyster *Saccostrea glomerata* (Andrew et al. 2010) exposed to estrogens (E2 and/or EE2) under laboratory conditions, and in intersex clams, *S. plana* (Ciocan et al. 2012). In contrast*, MeER1* and *MeER2* expressions were significantly downregulated in trochophore (early life) stages of the mussel *M. galloprovincialis* at 24 h post E2 (10 μg/L) and BPA (10 μg/L) exposure, although up-regulated at a lower dose of BPA (1 μg/L), suggesting dose dependent response (Balbi et al. 2016). With respect to both the *V9* and *ER2* expressions and implications of their change, it is important to consider the ongoing debate surrounding the role of estrogens in bivalve reproduction and any potential endocrine disruption. Molluscs contain vertebrate-like steroids, including E1 and E2 (Reis-Henriques et al. 1990; Zhu et al. 2003) and have steroidogenesis pathway enzymes (Janer and Porte 2007). Sex steroid presence is not doubted, but the biological role and possible disruption is debated (Scott 2013).

Linking to relevant biological endpoints of reproductive impact, *Bcl-2* (a regulator of cell death) and *fas* (encoding a protein ligand that binds to an associated receptor triggering apoptosis) expression have both been used as markers of apoptosis, including follicular atretic conditions, in vertebrates (Lee et al. 1997; Morita et al. 1999). This is the first instance of their use with *Mytilus* sp. Similar to *V9*, sex also influences *Bcl-2* mRNA expression levels significantly (SRH *p* = 0.037), but sampling site does not at QD in April; no interaction was detected between sex and site. In April, *Bcl-2* expression in females showed significant down-regulation relative to males from QD-B (Fig. 2C), which corresponded with the highest incidence of atresia (26.6%) in females at any site. *Bcl-2* expression inhibits oocyte follicle atresia in mice (Morita et al. 1999), thus a down-regulation in QD-B females may reflect the higher atretic incidence. In contrast, males collected in summer from YT and SS displayed significantly down regulated *Bcl-2* expression relative to females (Fig. 2C). In other studies, experimental E2 exposure significantly increases, while testosterone inhibits, *Bcl-2* expression (Huber et al. 1999), which corresponds with mussels from YT, then SS, having the highest tissue levels of total estrogens (Table S5). A significant increase in *Bcl-2* expression was also observed between July and April for QD-D males (Fig. 2C).

Increased *fas* expression has previously been associated with increased apoptosis in vertebrate gonadal cells (Lee et al. 1997; D’Alessio et al. 2001). Herein, *fas* expression levels were significantly influenced by sex (SRH, *p* = 0.023), but not sampling site (QD-A to D) in mussels during April, with no interaction detected between the two factors. *Fas* expression in males was significantly up-regulated relative to females sampled at QD-B during April, with a similar trend for QD-A and QD-D in April (Fig. 2D). *Fas* expression was also significantly higher in males at QD-D in April relative to July. In other species, experimental monophthalate exposure using mice has been shown to increase *fas* expression and trigger sertoli cell apoptosis (D’Alessio et al. 2001). Here, the aqueous BPA levels were relatively high at SS (28.36 ng/L, Table S5), yet no *fas* mRNA expression was observed in either sex (Fig. 2D).

The aqueous estrogen levels detected herein are relatively low (< LOD-28.36 ng/L, Table S5) compared with previous values (~ 1–200 ng/L range) from the region, and more in line with values (~ 1–30 ng/L) from other worldwide coastal locations (Table S1). Similarly, the tissue levels of total estrogens detected (Table S5) are generally lower (at ~ 0.5–3 ng/g ww) than those previously reported in Chinese coastal waters (1374–3199 ng/g lipid weight) (Zhang et al. 2011), though differing units make direct comparison problematical. The average estrogen levels (~ E2 1.94 ng/g, EE2 1.05 ng/g ww) (Table S5) in gonads are similar to Baltic Sea mussels *M. edulis-trossulus* hybrids (3.9–4.8 ng/g wet weight) (Zabrzanska et al. 2015) and Antarctic clams for E2 (0.8–2 ng/g wet weight) (Emnet et al. 2015). To compare, freshwaters receive effluents with EE2 as high as 800 ng/L (Koplin et al. 2002).

The East China coastal region is a receiving environment for many classes of contaminants (Table S1). Herein, the differential expression of reproduction-relevant, estrogen-specific biomarkers *V9* and *ER2*, indicate a potential biological impact related to (xeno)estrogen contaminants. Future work is required to further optimise and validate these biomarker responses, as well as monitor the reproduction endpoints of mussels to determine any population level impacts.

## Supplementary Information

Below is the link to the electronic supplementary material.

Supplementary file1 (DOCX 1741 kb)

## References

[CR1] Aarab N, Minier C, Lemaire S, Unruh E, Hansen PD, Larsen BK, Andersen OK, Narbonne JF (2004). Biochemical and histological responses in the mussel (*Mytilus edulis*) exposed to North Sea oil and to a mixture of North Sea oil and alkylphenols. Mar Environ Res.

[CR2] Andrew MN, O’Connor WA, Dunstan RH, MacFarlane GR (2010). Exposure to 17 alpha ethynylestradiol causes dose and temporally dependent changes in intersex, females and vitellogenin production in the Sydney rock oyster. Ecotoxicol.

[CR3] Arukwe A, Knudsen FR, Goksøyr A (1997). Fish zona radiata (eggshell) protein: a sensitive biomarker for environmental estrogens. Environ Health Perspect.

[CR4] Atkinson S, Atkinson M, Tarrant AM (2003). Estrogens from sewage in coastal marine environments. Environ Health Perspect.

[CR5] Balbi Y, Franzellitti S, Fabbri R, Montagna M, Fabbri R, Canesi C (2016). Impact of bisphenol A (BPA) on early embryo development in the marine mussel *Mytilus galloprovincialis*: effects on gene transcription. Environ Pollut.

[CR6] Barber BJ (2004). Neoplastic diseases of commercially important marine bivalves. Aquat Living Resour.

[CR7] Beninger PG (2017). Caveat observator: the many faces of pre-spawning atresia in marine bivalve reproductive cycles. Mar Biol.

[CR8] Beyer J, Green NW, Brooks S, Allan IJ, Ruus A, Gomes T, Bråte ILN, Schøyen M (2017). Blue mussels (*Mytilus edulis* spp.) as sentinel organisms in coastal pollution monitoring: a review. Mar Environ Res.

[CR9] Bustin SA, Benes V, Garson JA, Hellemans J, Huggett J, Kubista M, Mueller R, Nolan T, Pfaffl MW, Shipley GL, Vandesompele J, Wetter CT (2009). The MIQE guidelines: minimum information for publication of quantitative real-time PCR experiments. Clin Chem.

[CR10] Chen X, Li VWT, Yu RMK, Cheng SH (2008). Choriogenin mRNA as a sensitive molecular biomarker for estrogenic chemicals in developing brackish medaka. Ecotoxicol Environ Saf.

[CR11] Ciocan CM, Cubero-Leon E, Puinean AM, Hill EM, Minier C, Osada M, Fenlon K, Rotchell JM (2010). Effects of estrogen exposure in mussels, *Mytilus edulis*, at different stages of gametogenesis. Environ Pollut.

[CR100] Ciocan CM, Cubero-Leon E, Minier C, Rotchell JM (2011) Identification of reproduction-specific genes associated with maturation and estrogen exposure in a marine bivalve Mytilus edulis. PLoS ONE 6(7):e22326 10.1371/journal.pone.0022326 PMC3144882 21818309

[CR12] Ciocan CM, Cubero-Leon E, Langston WJ, Pope N, Peck MR, Minier C, Rotchell JM (2012). Intersex in *Scrobicularia plana*: transcriptomic analysis reveals novel genes involved in endocrine disruption. Environ Sci Technol.

[CR13] Cubero-Leon E, Ciocan CM, Hill EM, Osada M, Kishida M, Itoh N, Kondo R, Minier C, Rotchell JM (2010). Estrogens disrupt serotonin receptor and cyclooxygenase mRNA expression in the gonads of mussels (*Mytilus edulis*). Aquat Toxicol.

[CR14] Cubero-Leon E, Ciocan CM, Minier C, Rotchell JM (2012). Reference gene selection for qPCR in mussel, *Mytilus edulis*, during gametogenesis and exogenous estrogen exposure. Environ Sci Pollut Res.

[CR15] D’Alessio A, Riccioli A, Lauretti P, Padula F, Muciaccia B, De Cesaris P, Filippini A, Nagata S, Ziparo E (2001). Testicular fasL is expressed by sperm cells. Proc Natl Acad Sci USA.

[CR17] Ding J, Chen S, Qu M, Wang Y, Di Y (2020). Trophic transfer affects cytogenetic and antioxidant responses of the mussel *Mytilus galloprovincialis* to copper and BaP. Mar Environ Res.

[CR18] Emnet P, Gaw S, Northcott G, Storey B, Graham L (2015). Personal care products and steroid hormones in the Antarctic coastal environment associated with the two Antarctic research stations, McMurdo Station and Scott Base. Environ Res.

[CR20] Huber SS, Kupperman J, Newell MK (1999). Estradiol prevents and testosterone promotes fas dependent apoptosis in CD4+ Th2 cells by altering Bcl 2 expression. Lupus.

[CR21] Janer G, Porte C (2007). Sex steroids and potential mechanisms of non-genomic endocrine disruption in invertebrates. Ecotoxicol.

[CR22] Kaptaner B, Unal G (2011). Effects of 17 alpha-ethynlestradiol and nonylphenol on liver and gonadal apoptosis and histopathology in *Chalcalburnus tarichi*. Environ Toxicol.

[CR23] Kimball DM (1996). Reproductive pathology in *Mytilus edulis* from Boston Harbor and Cape Cod Bay. Mar Environ Res.

[CR24] Kiss T (2010). Apoptosis and its functional significance in molluscs. Apoptosis.

[CR25] Koplin DW, Furlong ET, Meyer MT, Thurman EM, Zaugg SD, Barber LB, Buxton HT (2002). Pharmaceuticals, hormones, and other organic wastewater contaminants in U.S. steams 1999–2000: a national reconnaissance. Environ Sci Technol.

[CR26] Koyama J, Kitoh A, Nakai M, Kohno K, Tanaka H, Uno S (2013). Relative contribution of endocrine-disrupting chemicals to the estrogenic potency of marine sediments of Osaka Bay, Japan. Water Air Soil Pollut.

[CR27] Lee J, Richburg JH, Younkin SC, Boekelheide K (1997). The fas system is a key regulator of germ cell apoptosis in the testis. Endocrinology.

[CR28] Livak KJ, Schmittgen TD (2001). Analysis of relative gene expression data using real-time quantitative PCR and the 2(T)(-Delta Delta C) method. Methods.

[CR29] Matozzo V, Marin MG (2005). 4-Nonylphenol induces immunomodulation and apoptotic events in the clam *Tapes philippinarum*. Mar Ecol Prog Ser.

[CR30] Morita Y, Perez GI, Maravei DV, Tilly KI, Tilly JL (1999). Targeted expression of Bcl-2 in mouse oocytes inhibits ovarian follicle atresia and prevents spontaneous and chemotherapy-induced oocyte apoptosis in vitro. Mol Endocrinol.

[CR31] Nagasawa K, Treen N, Kondo R, Otoki Y, Itoh N, Rotchell JM, Osada M (2015). Molecular characterisation of an estrogen receptor and estrogen-related receptor and the autoregulatory capabilities in two *Mytilus* species. Gene.

[CR32] Ortiz-Zarragoitia M, Cajaraville MP (2010). Intersex and oocyte atresia in the mussel population from the Biosphere’s Reserve of Urdaibai (Bay of Biscay). Ecotoxicol Environ Saf.

[CR33] Ortiz-Zarragoitia M, Garmendia L, Barbero MC, Serrano T, Marigomez I, Cajaraville MP (2011). Effects of the fuel oil spilled by the Prestige tanker on reproduction parameters of wild mussel populations. J Environ Monit.

[CR34] Osada M, Takamura T, Sato H, Mori K (2003). Vitellogenin synthesis in the ovary of scallop, control by estradiol-17α and the central nervous system. J Exp Zool.

[CR35] Park JJ, Shin YK, Hung SSO, Romano N, Cheon Y-P, Kim JW (2015). Reproductive impairment and intersexuality in *Gomphina veneriformis* by the tributyltin compound. Anim Cells Syst.

[CR36] Pope ND, Childs K, Dang C, Davey MS, O’Hara SCM, Langston K, Minier C, Pascoe PL, Shortridge E, Langston WJ (2015). Intersex in the clam *Scrobicularia plana* (Da Costa): Widespread occurrence in English Channel estuaries and surrounding areas. Mar Pollut Bull.

[CR37] Reis-Henriques MA, Le Guellec D, Remy-Martin JP, Adessi GL (1990). Studies of endogenous steroids from the marine mollusc *Mytilus edulis* L. by gas chromatography and mass spectrometry. Comp Biochem Physiol B.

[CR38] Scott AP (2013). Do mollusks use vertebrate sex steroids as reproductive hormones? II. Critical review of the evidence that steroids have biological effects. Steroids.

[CR39] Seed R (1969). The ecology of *Mytilus edulis* L. on exposed rocky shores. Oecologia.

[CR40] Seed R, Suchanek TH (1992) Population and community ecology of *Mytilus*. In: Gosling EM (ed) The mussel *Mytilus*: ecology, physiology, genetics and culture, 528 Amsterdam: Elsevier, pp 79–169

[CR41] Shi X, Zhou JL, Zhao H, Hou L, Yang Y (2014). Application of passive sampling in assessing the occurrence and risk of antibiotics and endocrine disrupting chemicals in the Yangtze Estuary, China. Chemosphere.

[CR42] Smolarz K, Hallmann A, Zabrzanska S, Pietrasik A (2017). Elevated gonadal atresia as biomarker of endocrine disruptors: field and experimental studies using *Mytilus trossulus* (L.) and 17-alpha ethinylestradiol (EE2). Mar Poll Bull.

[CR43] Sridevi P, Chaitanya RK, Prathibha Y, Balakrishna SL, Dutta-Gupta A, Senthilkumaran B (2015). Early exposure of 17α–ethynlestradiol and diethylstilbestrol induces morphological changes and alters ovarian steroidogenic pathway enzyme gene expression in catfish (*Clarias gariepinus*). Environ Toxicol.

[CR44] Suarez MP, Alvarez C, Molist P, San Juan F (2005). Particular aspects of gonadal cycle and seasonal distribution of gametogenic stages in *Mytilus galloprovincialis* cultured in the estuary of Vigo. J Shellfish Res.

[CR45] Qu M, Ding J, Wang Y, Chen S, Zhang Y, Di Y (2019). Genetic impacts induced by BaP and Pb in *Mytilus coruscus*: Can RAPD be a validated tool in genotoxicity evaluation both in vivo and in vitro?. Ecotoxicol Environ Saf.

[CR46] Vaschenko MA, Hsieh HL, Radashevsky VI (2013). Gonadal state of the oyster *Crassostrea angulata* cultivated in Taiwan. J Shellfish Res.

[CR47] Weber LP, Balch GC, Metcalfe CD, Janz DM (2004). Increased kidney, liver, and testicular cell death after chronic exposure to 17 alpha-ethinylestradiol in medaka (*Oryzias latipes*). Environ Toxicol and Chem.

[CR48] Yan C, Yang Y, Zhou J, Liu M, Nie M, Shi H, Gu L (2013). Antibiotics in the surface water of the Yangtze Estuary: occurrence, distribution and risk assessment. Environ Pollut.

[CR49] Ye A, Yang Y, Zhang J, Liu M, Hou LJ, Zhou JL (2013). Simultaneous determination of steroidal and phenolic endocrine disrupting chemicals in fish by ultra-high-performance LCMS/MS. J Chromatogr A.

[CR50] Zabrzanska S, Smolarz K, Hallman A, Konieczna L, Baczek T, Wolowicz M (2015). Sex related differences in steroid concentrations in the blue mussel (*M. edulis trossulus*) from the southern Baltic Sea. Comp Biochem Physiol A.

[CR51] Zhang X, Gao YJ, Li QZ, Li GX, Guo QH, Yan CZ (2011). Estrogenic compounds and estrogenicity in surface water, sediments, and organisms from Yundang Lagoon in Xiamen, China. Arch Environ Contam Toxicol.

[CR52] Zhu W, Mantione K, Jones D, Salamon E, Cho JJ, Cadet P (2003). The presence of 17-beta estradiol in *Mytilus edulis* gonadal tissues: evidence for estradiol isoforms. Neuro Endocrinol Lett.

