# Supplementary material for: Gonadal Atresia, Estrogen-Responsive, and Apoptosis-Specific mRNA Expression in Marine Mussels from the East China Coast: A Preliminary Study
Source: Bull Environ Contam Toxicol. 2022 Jan 24;108(6):1111–7. doi: 10.1007/s00128-022-03461-2 (PMC9188513; doi:10.1007/s00128-022-03461-2)
Supplement: Supplementary file 1 — Supplementary file1 (DOCX 1741 kb) [file 128_2022_3461_MOESM1_ESM.docx]

**Supplementary Information Cover Sheet**

Authors: Jingmin Zhu, Jiana Li, Emma Chapman, Huahong Shi, Corina M. Ciocan, Kai Chen,

Xiaodong Shi, JunLiang Zhou, Peiying Sun, Yueyao Zheng, and Jeanette M. Rotchell

Manuscript title: Gonadal atresia, estrogen-responsive, and apoptosis-specific mRNA expression in marine mussels from the East China coast: a baseline study.

Summary: 9 pages, 5 Tables, 1 Figure, and additional references.

**Supplementary Information Table and Figure Legends**

**Table S1**. Summary of work published on the concentrations and biological impacts of pollutant classes in the Chinese coastal region.

**Table S2**. Biometric data and sex ratios for mussels. *Significantly different from unity, Chi squared test. Abbreviations of sampling sites: QD, Qingdao; SS, Shengsi; XM, Xiamen; YT, Yantai.

**Table S3**. PCR primers used and amplicon sizes.

**Table S4**. Stages of gametogenesis for the mussels sampled at each location

**Table S5**. Estrogen concentrations for water samples and mussel tissues at each sampling site

**Figure S1**. Sampling site locations.

Table S1.

| Sample source | Location, year | Contaminant concentrations | Biological effects | Reference |
| --- | --- | --- | --- | --- |
| Mussel tissue,  *M.edulis* & *Perna viridis* | East Coast of China 2001 | Metals: Ag, As, Cd, Cr, Ni, Pb, Se, Zn, Cu, Fe and Hg – 0.14, 26.76, 5.31, 15.72, 4.78, 2.93, 7.40, 231, 54.17, 1002, and 317.3 μg/g dw  DDT: 14-640 μg/g dw  PAHs: 456-3495 μg/g dw  PCBs: 1-13 ng/g dw  PHCs: 621-2863 μg/g dw  Chongming Dao, Shanghai & Qingdao highest concentrations | Not studied | Fung et al. 2004 |
| Water | Yangzte River and Estuary, China | EDCs: nonylphenol (NP), 13-186 ng/L; octylphenol (OP), 4-96 ng/L; diethylstilbestrol, 2-14 ng/L; E1, 18-48 ng/L; E2, 6-24 ng/L | Not studied | Ping, 2011 |
| Water and sediments | Xiamen Bay, China | E2, EE2, diethystilbesterol, NP, OP, BPA. Total estrogens 49-1231 ng/g dw sediment and 102.33-4376.60 ng/L pore water | Not studied | Zhang et al. 2009 |
| Water, sediments and marine organisms | Yundang Lagoon, Xiamen, China | E2, EE2, diethystilbesterol, NP, OP, BPA. Total estrogens 609-711 ng/L surface water, 562-1038 ng/L pore water, 1433-2060 ng/g in sediments, 1374-3199 ng/g lipid weight in biota | Not studied | Zhang et al. 2011 |
| Water | Pearl River Estuary | NP, BPA, EE2, E2, E3 in the ranges of 0.2-178 ng/L | Not studied | Xu et al. 2014 |
| Water and fish tissues | Chongqing, China | NPs, total levels 1-98 μg/L in April | Not studied | Shao et al. 2005 |
| Water and sediments | Daliao River Estuary, China | NPs including BPA. Total levels 83.6-777 ng/L water and 1.5-456 ng/g dw sediment | Not studied | Li et al. 2013 |
| Marine species (n=14) | Bohai Bay, China | NPs | Trophic magnification shown | Hu et al. 2005 |
| Sediments | Yangtze Estuary China | Radionuclides: Pu, 0.8 mBq/g; ^137^Cs, 5 mBq/g | Not studied | Liu et al. 2011 |
| Water, sediments and marine organisms | Entire China coastal region | Metals in tissues: Cd, 0.4-49.5 μg/g dw; Hg, 0.04-1.36 μg/g dw | Not studied | Pan and Wang, 2012 |
| Water, sediment | Yangtze Estuary China | Pharmaceuticals (in water ng/L): propranolol 0.3-142, sulfamethoxazole 4.2-765, mebeverine 0-71, carbamazepine 17-675, tamoxifen 120-224, indomethacine 159-979, diclofenac 0-843, and meclofenamic acid 0-679. | Not studied | Yang et al. 2011 |
| Water, sediment | China, a review article | Pharmaceuticals and personal care products | Not studied | Bu et al. 2013 |
| *Crassostrea* sp. | Northern Yellow Sea of China | Organochlorine pesticides: aHCH 0.91-13.92 ng/g, aDDT 10-411 ng/g | Not studied | Chen et al. 2014 |
| Marine species | Northern Yellow Sea of China | PBDEs: 0.23-11 ng/g dw | Not studied | Chen et al. 2013 |
| Marine species | Liaodong Bay, North China | PBDEs 0.87-91.4 ng/g | Not studied | Ma at el. 2013 |
| Water, sediments and marine organisms | Xiamen coastal region, China | PBDEs 0.27-76.54 ng/g sediment, 2.5-34.1 ng/L in pore water, 0.33-1.26 ng/g lipid weight in biota (fish, clam, crab) | Not studied | Li et al. 2010 |
| Sediment and marine organisms | Xiamen coastal region, China | PCBs 2.33-30.94 ng/g | Not studied | Li et al. 2011 |
| Transplanted mussel species (n=3) and semipermeable membrane devices (SPMD) | Entire China coastal region | In mussels from polluted sites:  4-NP, 99.4 ± 9.40 to 326.1 ± 3.16 ng/g dry wt.  BPA, 170.3 ± 4.00 to 437.2 ± 36.8 ng/g dry wt.  E2, 82.9 ± 3.03 to 315.6 ± 6.50 ng/g dry wt.  EE2, 124.5 ± 9.26 to 204.5 ± 9.26 ng/g dry wt.  Strong, positive correlation between strong, positive correlation between EDC levels in transplanted mussels and SPMDs. | Not studied | Chiu et al., 2018 |
| Water, mussels | East China coastal region | E1 0.3-0.93, E2 <LOD-0.11, E3 <LOD-1.24, EE2 <LOD-0.1, BPA 0.97-28.36 ng/L in water.  Total E: 0.42-3.34 ng/g wet weight | Atresia incidence, sex ratio skew, gene expression changes | This study |
| **Worldwide values for estrogens in coastal regions** | | | | |
| Water | Acushnet River Estuary, USA | E1 0.8, E2 0.8, EE2 4.7 ng/L | Not studied | Zuo et al. 2006 |
| Water | Key Largo Harbor, USA | E1 5.2, E2 1.8, BPA 32 ng/L | Not studied | Singh et al. 2010 |
| Water | Netherlands, coastal sites | E1 3.4, E2 2.8, EE2 <LOD ng/L | Not studied | Belfroid et al. 1999 |
| Water | Osaka Bay, Japan | E1 0.1-0.9, E2 0.1-1.57, BPA <LOD-7.1 ng/L | Not studied | Koyama et al. 2013 |
| Water, clam *Laternula elliptica* | Erebus Bay, Antarctica | E2 0.8-2.0 ng/g, EE2 1.5-4.3 ng/g wet weight | Not studied | Emnet et al. 2015 |
| Water, tissues | Pacific Ocean to East Coast USA survey | Open ocean: E1 0.5 ng/L  Rehoboth Bay, Delaware: E1 1.87 ng/L | Not studied | Atkinson et al. 2003 |
| Sediments | Entire Chile coastal region | E1 0.06-4.61, E2 0.06-16.81, EE2 4.18-48.14 ng/g dw | Not Studied | Bertin et al. 2011 |
| Mussels (gonad and gill tissues) | Baltic Sea | Average tissue levels for both sexes combined: gonad E1 2.34, E3 2.03 ng/g ww; testosterone gill 15.3, gonad 5.3 ng/g ww  Average tissue levels: E2 testis 4.81 and ovary 3.86 ng/g ww | Normal sex ratio and gameto-genesis | Zabrzanska et al. 2015 |

**Table S2**.

| Sample site | Total no. collected | Mean length ± SEM (mm) | Histology determined Sex (n=) | | | | Females with atresia (%) | qPCR (n=) | |
| --- | --- | --- | --- | --- | --- | --- | --- | --- | --- |
|  |  |  | m | f | herm | spent |  | m | f |
| ***April, 2014*** | | | | | | | | | |
| QD-A | 30 | 38.3±2.5 | 18 | 12 | 0 | 0 | 6.6 | 16 | 12 |
| QD-B | 30 | 46.5±3.4 | 14 | 16 | 0 | 0 | 26.6 | 11 | 14 |
| QD-C | 30 | 44.7±3.4 | 15 | 15 | 0 | 0 | 13.3 | 12 | 14 |
| QD-D | 30 | 37.5±2.6 | 22 | 8 | 0 | 0 | 16.6 | 17 | 5 |
| ***July, 2014*** | | | | | | | | | |
| YT | 58 | 46.5±5.6 | 9 | 20 | 1 | 28 | 0 | 8-9 | 12-14 |
| QD-B | 28 | 42.6±2.6 | 1 | 6 | 0 | 21 | 0 | nd | 4-5 |
| QD-D | 30 | 46.1±2.9 | 9 | 8 | 0 | 13 | 0 | 6 | nd |
| SS | 60 | 36.4±4.2 | 27 | 32 | 1 | 0 | 0 | 10-14 | 11-18 |
| XM | 26 | 38.0±7.2 | 15 | 11 | 0 | 0 | 0 | 4-5 | 5-7 |

Table S3.

| **Gene** | **GenBank no.** | **Forward primer (5’-3’)** | **Reverse primer (5’-3’)** | **Size (bp)** | **PCR**  **efficiency (%)** |
| --- | --- | --- | --- | --- | --- |
| *18S* | L33448 | GTGCTCTTGACTGAGTGTCTCG | CGAGGTCCTATTCCATTATTCC | 116 | 98.1 |
| *EF1* | AY580270 | CACCACGAGTCTCTCCCAGA | GCTGTCACCACAGACCATTCC | 105 | 100.3 |
| *V9* | HQ664949 | TTCTGGACGAAATGCTAATGTGA | GGATTGAGCGTGACGAGACC | 90 | 92.8 |
| *ER2* | AB257133 | GGAACACAAAGAAAAGAAAGGAAG | GCTGGATTAGGACTGCCACTTG | 116 | 98.9 |
| *Bcl2* | KC545829 | GACAGTCCGTGGGATGTGAA | CTTAACGCCATTGCGCCTAT | 71 | 99.3 |
| *Fas* | KF051276 | ACGCTGATGACATACATCGCA | CGGTACTGGCTGAGCTTGTT | 311 | 97.6 |

Table S4

| **Sampling site** | **Female %** | | | **Male %** | | | **Non differentiated**  **%** |
| --- | --- | --- | --- | --- | --- | --- | --- |
|  | Developing stages (β) | Spawning stages (γ) | Resting stage (γI) | Developing stages | Spawning stages | Resting stages | Spent |
| ***April, 2014*** |  |  |  |  |  |  |  |
| QD-A | 10 | 30 | 0 | 46.6 | 13.3 | 0 | 0 |
| QD-B | 13.3 | 40 | 0 | 10 | 36.6 | 0 | 0 |
| QD-C | 16.6 | 30 | 3.3 | 36.6 | 13.3 | 0 | 0 |
| QD-D | 13.3 | 13.3 | 0 | 10 | 63.3 | 0 | 0 |
| ***July, 2014*** |  |  |  |  |  |  |  |
| YT | 1.6 | 0 | 33.6 | 13.3 | 0 | 3.3 | 48 |
| QD-B | 7.2 | 0 | 14.3 | 3.5 | 0 | 0 | 75 |
| QD-D | 10 | 3.3 | 13.3 | 23.3 | 3.3 | 3.3 | 43.3 |
| SS | 20 | 33.3 | 0 | 30 | 16.6 | 0 | 0 |
| XM | 38.5 | 3.8 | 0 | 19.2 | 38.5 | 0 | 0 |

Table S5.

| *Sample site | Aqueous estrogen levels (ng/L) | | | | | Tissue estrogen levels (ng/g wet weight) | | | | |
| --- | --- | --- | --- | --- | --- | --- | --- | --- | --- | --- |
|  | E1 | E2 | E3 | EE2 | BPA | E1 | E2 | E3 | EE2 | BPA |
| ***April, 2014*** |  |  |  |  |  |  |  |  |  |  |
| QD-A | 0.74 | <LOD | <LOD | <LOD | 10.85 | nd | nd | nd | nd | nd |
| QD-B | 0.76 | <LOD | <LOD | <LOD | 15.63 | nd | nd | nd | nd | nd |
| QD-C | 0.76 | <LOD | <LOD | <LOD | 5.66 | nd | nd | nd | nd | nd |
| QD-D | 0.72 | <LOD | <LOD | <LOD | 5.54 | nd | nd | nd | nd | nd |
| ***July, 2014*** |  |  |  |  |  |  |  |  |  |  |
| YT | 0.58 | 0.11 | <LOD | <LOD | 9.2 | 0.61±0.04 | 1.21±0.03 | 0.91±0.02 | 0.61±0.1 | 0.3±0.02 |
| QD-B | 0.3 | <LOD | <LOD | 0.1 | 0.97 | nd | nd | nd | nd | nd |
| QD-D | 0.4 | <LOD | <LOD | <LOD | 1.51 | <LOD | <LOD | <LOD | <LOD | 1.04±0.02 |
| SS | 0.36 | <LOD | <LOD | 1.24 | 28.36 | 0.22±0.01 | 0.54±0.08 | 0.43±0.01 | 0.33±0.1 | 3.88±0.07 |
| XM | 0.93 | 0.11 | <LOD | <LOD | 9.87 | >LOD | 0.19±0.03 | 0.25±0.06 | <LOD | 3.27±0.04 |

*Note: Abbreviations of sampling sites: QD, Qingdao; SS, Shengsi; XM, Xiamen; YT, Yantai.

nd –no data

Fig. S1.


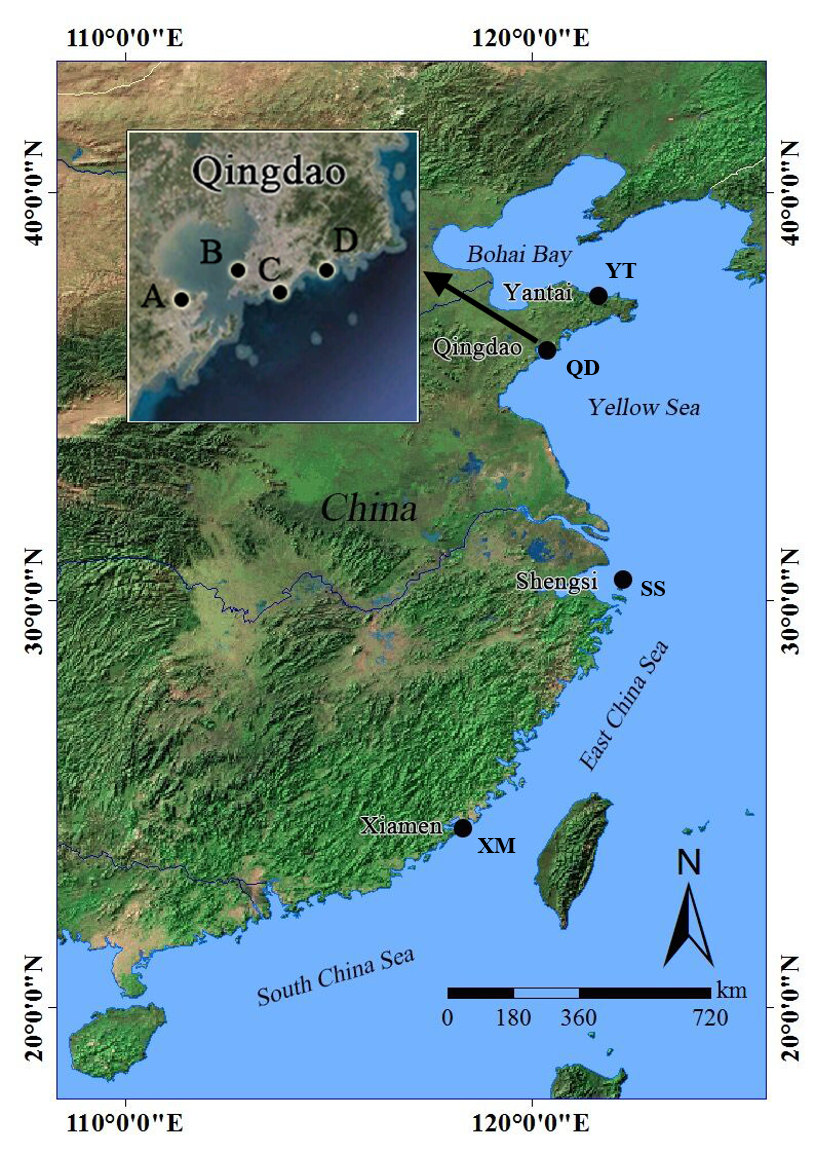


**Supplementary information - additional references contained in Table S1.**

Bu QW, Wang B, Huang J, Deng SB, Yu G (2013) Pharmaceuticals and personal care products in the aquatic environment in China: A review. J Hazard Mater 262: 189-211.

Chen J, Chen LL, Liu D, Zhang G (2013) Polybrominated diphenyl ethers contamination in marine organisms of Yantai coast, Northern Yellow Sea of China. Bull Environ Contam Toxicol 90: 679-683.

Chen J, Chen LL, Liu DY, Zhang GS (2014) Organochlorine pesticide contamination in marine organisms of Yantai coast, northern Yellow Sea of China. Environ Monit Assess 186: 1561-1568.

Chiu JM, Po BH, Degger N, Tse A, Liu W, Zheng G, Zhao DM, Xu D, Richardson B and Wu RS (2018) Contamination and risk implications of endocrine disrupting chemicals along the coastline of China: A systematic study using mussels and semipermeable membrane devices. Sci Total Environ 624: 1298-1307.

Fung CN, Lam JCW, Zheng GJ, Connell DW, Monirith I, Tanabe S, Richardson BJ, Lam PKS (2004) Mussel-based monitoring of trace metal and organic contaminants along the East

Coast of China using *Perna viridis* and *Mytilus edulis*. Environ Pollut 127: 203-216.

Hu JY, Jin F, Wan Y, Yang M, An LH, An W, Tao S (2005) Trophodynamic behavior of

4-monylphenol and nonylphenol polyethoxylate in a marine aquatic food web from Bohai Bay, North

China: Comparison to DDTs. Environ Sci Technol 39: 4801-4807.

Li QZ, Yan CZ, Luo ZX, Zhang X (2010) Occurrence and levels of polybrominated diphenyl ethers (PBDEs) in sediments and marine organisms from Xiamen offshore areas, China. Mar Pollut Bull 60: 464-469.

Li QZ, Luo ZX, Yan CZ, Zhang X (2011) Assessment of polychlorinated biphenyls contamination in sediment and organism from Xiamen offshore area, China. Bull Environ Contam Toxicol 87: 372-376.

Li Z, Gibson M, Liu C, Hu H (2013) Seasonal variation of nonylphenol concentrations and fluxes with influence of flooding in the Daliao River Estuary, China. Environ Monit Assess 185: 5221-5230.

Liu Z, Zheng J, Pan S, Dong W, Yamada M, Aono T, Guo Q (2011) Pu and ^137^Cs in the

Yangtze River Estuary sediments: distribution and source identification. Environ Sci Technol 45: 1805-1811.

Ma XD, Zhang HJ, Yao ZW, Zhao XF, Wang LX, Wang Z, Chen JP, Chen JW (2013) Bioaccumulation and trophic transfer of polybrominated diphenyl ethers (PBDEs) in a marine food web from Liaodong Bay, North China. Mar Poll Bull 74: 110-115.

Pan K, Wang WX (2012) Trace metal contamination in estuarine and coastal environments in

China. Sci Total Environ 421-422: 3-16.

Ping XY (2011) Distribution characteristics of five kinds of endocrine disrupting chemicals in the

Yangtze River Estuary and adjacent areas. International Conference on Remote Sensing, Environment and Transportation Engineering. 7486-7489.

Shao B, Hu JY, Yang M, An W, Tao S (2005) Nonylphenol and nonylphenol ethoxylates in river water, drinking water, and fish tissues in Chongqing, China. Arch Environ Contam Toxicol 48: 467-473.

Singh SP, Azua A, Chaudhary A, Khan S, Willett K, Gardinali PR (2010) Occurrence and distribution of steroids, hormones and selected pharmaceuticals in South Florida coastal environments.

Ecotoxicol 19: 338-350.

Xu W, Yan W, Huang W, Miao L, Zhong L (2014) Endocrine-disrupting chemicals in the Pearl River Delta and coastal environment: sources, transfer and implications. Environ Geochem Health 36: 1095-1104.

Yang Y, Fu J, Peng H, Hou L, Liu M, Zhou JL (2011) Occurrence and phase distribution of

elected pharmaceuticals in the Yangtze Estuary and its coastal zone. J Hazard Mater 190: 588-596.

Zhang X, Li QZ, Li GX, Wang ZS, Yan CZ (2009) Levels of estrogenic compounds in

Xiamen Bay sediment, China. Mar Pollut Bull 58: 1210-1216.

Zuo YG, Zhang K, Deng YW (2006) Occurrence and photochemical degradation of 17 alpha

ethinylestradiol in Acushnet River Estuary. Chemosphere 63: 1583-1590.
